# Job Demands and Resources, Burnout, and Psychological Distress of Social Workers in China: Moderation Effects of Gender and Age

**DOI:** 10.3389/fpsyg.2021.741563

**Published:** 2021-12-10

**Authors:** Xiaoxia Xie, Chienchung Huang, Shannon P. Cheung, Yuqing Zhou, Jingbo Fang

**Affiliations:** ^1^Research Institute of Social Development, Southwestern University of Finance and Economics, Chengdu, China; ^2^School of Social Work, Rutgers, The State University of New Jersey, New Brunswick, NJ, United States

**Keywords:** job demands, job resources, burnout, psychological distress, social workers, China

## Abstract

Social work is a fast-growing profession in China, with the workforce numbering approximately 1.2 million in 2018. Studies have shown, however, that social workers in China experience high burnout rates and significant psychological distress. Analyzing data collected from 897 social workers in Chengdu, China, we applied the job demands and resources (JD-R) theory to examine the effects of JD-R on burnout and psychological distress in social workers, as well as whether these relations are moderated by gender and age. Results supported a dual process by which JD-R affected both social workers’ burnout and psychological distress through health impairment and motivation processes. Job demands (JD) were associated with high burnout and psychological distress. Meanwhile, job resources (JR) were associated with reduced burnout and psychological distress. Results indicated that JR had greater effects on burnout and distress than did JD. Women and younger professionals appeared to be affected most by JD and psychological distress. The findings support a need for interventions that buffer the effects of JD-R on burnout and psychological distress in social workers in China, focusing on women and younger professionals.

## Introduction

### Social Work Development in China

Within the past century, social work in China has experienced major periods of emergence, suspension, and revival (Tang and Li, 2021). In response to growing social issues that accompanied the country’s rapid economic development since the 1980s, China has seen significant expansions to its social work labor force and education programs. Peking University first began recruiting undergraduate social work students in 1989. By the end of 2018, over 500 undergraduate, master’s, and Ph.D. programs had been established, together producing over 40,000 social work graduates each year. While the national social work labor force in China totaled 0.2 million in 2010, by 2018, it had grown to about 1.2 million (Li et al., 2012; Department of Civil Affairs, 2019; Chan et al., 2020). These numbers indicate that social work in China has experienced an unprecedented scale of expansion in a short period of time. Yet, the development of social work across the country varies, mirroring the availability of resources by location. In urban centers (e.g., Shanghai, Beijing, Guangzhou, and Shenzhen), which have greater resource availability, the profession has experienced greater development, as compared to rural regions in western China (Liu et al., 2020). Regardless of the rural-urban divide, the future of this field in China is threatened by high burnout and turnover rates (Jiang et al., 2019; Su et al., 2020; Tang and Li, 2021).

### Burnout and Social Work

Internationally, social workers and other human service workers experience substantial rates of burnout (Lizano, 2015; Sánchez-Moreno et al., 2015). The burnout and turnover rates for social workers are similarly high in China (Du, 2015; Zhu, 2015; Jiang and Wang, 2016; Wang et al., 2019). In 2014, 25% of social workers in Guangzhou had left their jobs (Zhu, 2015), and between 2008 and 2015, the percentage of social workers who left their positions in Shenzhen increased by approximately 10 percentage points, from 8.2 to 18.08% (Du, 2015). Meanwhile, in 2013, the turnover rate of social workers in Shenzhen was over 22% (Du, 2015). Cross-cultural research has supported that burnout is strongly associated with turnover (Abu-Bader, 2000; Barak et al., 2001; Lloyd et al., 2002; Evans et al., 2006; Kim and Stoner, 2008; Luo and Lei, 2021). This relation is prevalent among social workers in China as well (Wang et al., 2021). High rates of burnout and turnover have negative implications for the field of social work, its labor force, and its client populations. Social workers experiencing burnout may be unable to provide quality services to clients, which can harm the vulnerable populations that they serve. Moreover, high turnover rate may lead to increased job demands and workloads for those social workers who remain in the labor force, leading to further potential for burnout, which can in turn exacerbate the cycle of burnout and turnover. Although social workers in China are actively engaged in providing services to vulnerable populations across settings as diverse as schools, community centers, hospitals, and other social welfare organizations and agencies (Jiang et al., 2019; Chan et al., 2020), the profession’s high burnout and turnover rates raise concerns about the ability of social workers to sustain the provision of quality services. As such, it is imperative that scholars investigate the factors that contribute to and mitigate burnout, and associated psychological distress, in social work professionals in China. This study thus applies the job demands and resources (JD-R) theory to examine how job demands (JD) and job resources (JR) affect job outcomes of Chinese social workers. The findings of this study can (1) build knowledge of work conditions and psychological distress faced by social workers in China, an emerging profession with high burnout rates, (2) contribute to understanding whether the JD-R theory applies to this vulnerable population, and (3) shed light on potential interventions and services to mitigate the burnout and psychological distress that jeopardize the well-being of social workers in China.

### The Job Demands-Resources Theory, Burnout, and Psychological Distress

In the JD-R theory, proposed by Demerouti et al. (2001), working conditions are categorized into two groups, JD and JR, which differentially affect worker burnout and other occupational outcomes (Demerouti et al., 2001; Bakker and Demerouti, 2007; Lequeurre et al., 2013). JD include various aspects of the job (i.e., physical, social, or organizational) that require a sustained physical or mental effort. This effort comes at a physiological cost, such as exhaustion and fatigue. JR, on the other hand, are aspects of the job (i.e., psychological, social, or organizational) that can facilitate the achievement of work goals, mitigate the psychological cost of job demands, and/or stimulate personal growth and development (Demerouti et al., 2001). Resources are described as “health-protecting factors,” which keep individuals healthy, even after facing a significant workload (Demerouti et al., 2001, p. 501). The JD-R theory posits that JD and JR affect burnout, then psychological well-being, *via* two processes: the health-impairment process (also called the energy-driven process) and the motivation-driven process. In the former, through JD, workers experience a gradual depletion of their energy, leading to burnout and psychological distress. In the latter (the lack of), JR can prevent employees from fulfilling their role or from meeting their responsibilities, which, in turn, leads to frustration, withdrawal, and, ultimately, burnout, and distress (Demerouti et al., 2001; Bakker et al., 2003).

### JD-R and Burnout

Burnout, a psychological syndrome that involves emotional exhaustion, depersonalization, and a diminished sense of personal accomplishment, can occur while working in challenging situations (Maslach et al., 1996). Burnout has long been recognized as an occupational hazard for human service professionals (Aiken et al., 2002; Hakanen et al., 2006), including social workers (Travis et al., 2015). In fact, social workers are considered an occupational group that is at above-average risk of burnout (Soderfeldt et al., 1995).

Job demands and resources has been found to be important factors of burnout and job satisfaction in social workers and other human service professionals (Demerouti et al., 2001; Tang et al., 2017; Su et al., 2020). In studies with samples of Chinese social workers, including both newly recruited social workers and early stage professionals (Tang et al., 2017; Tang and Li, 2021), scholars have found evidence of a positive relation between JD and burnout and turnover, as well as a negative relation between JR and burnout (Su et al., 2020; Luo and Lei, 2021). Su et al. (2020), for example, applied the JD-R theory in an analysis of data from a nationally representative random sample of social workers (*n* = 5,800) and found that psychosocial resources, such as professional recognition and various types of social support, significantly reduced burnout. This relation has yet to be studied within the context of psychological distress, despite the possibility that burnout, as an experience of emotional exhaustion, depersonalization, and reduced sense of personal achievement (Maslach et al., 1996), may act as a stressor that precipitates psychological distress.

### JD-R and Psychological Distress

When faced with a stressor or a demand that is difficult to cope with, individuals may experience psychological distress, an emotional state of deep discomfort (Ridner, 2004; Arvidsdotter et al., 2016). Characterized by symptoms of depression and anxiety (Ridner, 2004), psychological distress has been shown to be related to JD-R (Oshio et al., 2018; Ben-Ezra and Hamama-Raz, 2021). Cross-cultural studies in both Japan (Shimazu et al., 2010; Oshio et al., 2018) and Israel (Ben-Ezra and Hamama-Raz, 2021) have supported a positive correlation between JD and psychological distress. While Shimazu et al. (2010) and Oshio et al. (2018) studied samples of Japanese adults who worked in a vast array of occupations, Ben-Ezra and Hamama-Raz (2021) specifically studied social workers who were working during the COVID-19 pandemic (*n* = 615). Results indicated that JD had a significant direct effect on psychological distress among the social workers (*r* = 0.233, *p* < 0.001).

Studies such as these, which examine the antecedents of psychological distress, are essential given that psychological distress has been found to positively predict serious mental illness, alcohol and substance use, and negative work outcomes (Furukawa et al., 2003; Hardy et al., 2003; Kessler et al., 2010). Mood disorders and anxiety disorders, for example, have been found to be positively associated with psychological distress (Furukawa et al., 2003). This is supported across a wide variety of cultural contexts. Analysis of data drawn from samples in 14 countries similarly revealed that psychological distress was a predictor of serious mental illness (Kessler et al., 2010).

Though it has been well-established that psychological distress varies by gender and age (Kilkkinen et al., 2007; Wong and Shobo, 2016; Zhang et al., 2018; Horesh et al., 2020), the moderating effects of gender and age on the relation between psychological distress and its antecedents are relatively understudied. Given the socio-cultural and health contexts that accompany both gender and age, investigating the relation between burnout and psychological distress by these two demographic characteristics can provide a better understanding of how different subgroups of social work professionals are affected by JD-R. This is critical to our understanding of the underlying mechanisms of burnout and psychological distress among social workers in China.

In short, the JD-R theory has been widely tested in and supported by studies conducted across a plethora of disciplines, including human services, industry, and transportation (Demerouti et al., 2001; Bakker et al., 2003; Luo and Lei, 2021), and country contexts (Hakanen et al., 2006; Hu et al., 2011; Lequeurre et al., 2013; Bakker et al., 2014; Kwon and Kim, 2020). Similarly, the JD-R model has been applied in and supported by studies that examine work outcomes such as burnout, stress, work engagement, and health (Hakanen et al., 2008; Schaufeli et al., 2009; Ren et al., 2013; Schaufeli and Taris, 2014; Grover et al., 2017). Findings underscore that JD play a significant role in the health-impairment process and the development of burnout. Meanwhile, JR, through the motivation process, can be a significant protective factor against burnout. The findings also support that burnout mediates the effects of JD-R on work and health outcomes (Hakanen et al., 2008; Schaufeli et al., 2009). Empirical studies have shown that JD-R have profound consequences on burnout and individual well-being. However, few have centered on the well-being of social workers in China. Further, whether gender and age moderate the relation between burnout and well-being has yet to be investigated. Thus, we apply the JD-R theory to examine the effects of JD-R on burnout and psychological distress and whether this relation is moderated by gender and age in a sample of Chinese social workers.

## Hypotheses

Based on the JD-R theory (Demerouti et al., 2001), we hypothesized a mediational pathway by which JD-R affects psychological distress *via* burnout. We also examined whether this pathway is moderated by gender and age, as past literature has indicated that distress varies by gender and age. Our hypotheses are as follows:

1. JD are positively associated with burnout, while JR are negatively associated with burnout.
2. Burnout and JD are positively associated with psychological distress, while JR are negatively associated with psychological distress.
3. Burnout is a mediator of the relation between JD, JR, and psychological distress.
4. The effects of JD and burnout on psychological distress are greater for women and younger social workers, compared with their respective counterparts.

## Materials and Methods

### Data and Sample

The data for the present study came from an online anonymous survey administered to social workers in Chengdu, China, the capital of Sichuan province and a city that has seen rapid development in social work (Department of Civil Affairs, 2019; Chengdu Department of Civil Affairs, 2020). We randomly selected two districts from 22 districts in Chengdu, then contacted local social workers associations and agencies to recruit participants. Each district had around 600 social workers. All social workers in the two districts were invited to complete the survey on May 5, 2021. Reminders to participate in the survey were sent to social workers 7 and 14 days after the initial invitation. Around 915 social workers participated in the online survey between May 5 and May 29 in 2021. About 18 surveys had incomplete answers and were therefore excluded from the final analysis. Our final analytic sample contained data from 897 social workers, indicating a response rate of 75%. Over three-quarters (78.2%) of the sample was female. The average age of the sample was 31.8 (*SD* = 7.3). Over half had at least a college degree (54.6%) as well as a social work license (52.3%).

The research protocol was approved by the research review committee at one of the co-authors’ university in China. An informed consent process was implemented prior to the survey. Respondents received 5 RMB (1 USD) as compensation for their participation. Participants were informed that their participation was voluntary and that they could choose to stop completing the survey at any time.

### Measures

#### Psychological Distress

The dependent variable, psychological distress, was assessed *via* the Kessler 6 Psychological Distress Scale (K6), developed by Kessler et al. (2003). The K6 has shown high validity and reliability in past studies (e.g., Kessler et al., 2010; Oshio et al., 2018; Twenge and Joiner, 2020). It consists of six questions that ask respondents about 30-day prevalence of psychological distress, such as nervousness, hopelessness, restlessness, depression, and worthlessness. Participants identified how frequently they felt each of these emotions, as well as how frequently they felt that “everything was an effort.” Possible responses ranged from 0, “none of the time,” to 4, “all of the time.” Responses to all items were summed up. Total scores could range from 0 to 24. Following past calibration studies (Kessler et al., 2002, 2003; Furukawa et al., 2003; Fushimi et al., 2012), total scores corresponded with one of three levels of psychological distress, indicated by the following score ranges: 13–24 (high); 8–12 (moderate); and 0–7 (low). In this study, the Cronbach’s alpha of the K6 scale was 0.94.

Burnout was assessed by the Maslach Burnout Inventory, Human Services Survey (MBI-HSS; Maslach et al., 1996). The survey consists of 22 items which measure a multidimensional concept of burnout. The psychometric soundness, reliability, and validity of the MBI-HSS have been verified in samples of working professionals of numerous occupations, languages, and countries (Naude and Rothmann, 2004; Aguayo et al., 2011; Chen et al., 2021). Zhang et al. (2006) used 22-item scale with a sample of approximately 4,850 Chinese police officers. Results of factor analysis showed that five items had high factor loadings across several multiple dimensions in the Chinese sample. These items were therefore removed from the scale. The remaining 17 items comprise the Chinese version of MBI-HSS, which has good reliability (Cronbach’s alpha of 0.71; Zhang et al., 2006). We used the Chinese version of MBI-HSS in the present study. The score for each item ranges from 0 (never) to 6 (every day). We reversed the score of positive items so that higher scores indicated higher levels of burnout. Possible scores ranged from 0 to 102. The Cronbach’s alpha of the Chinese version of MBI-HSS was 0.88 in this study.

We measured JD-R using a multidimensional scale from Questionnaire sur les Ressources et Contraintes Professionnelles (QRCP) of Lequeurre et al. (2013). Given the work of social workers in China, we focus on two dimensions of JD, workload and emotional workload, and two areas of JR, relationships with colleagues and information. Workload refers to the sense of having too much work to do in the time available, while emotional workload describes emotional JD that require respondents to expend energy to deal with job-related emotions (e.g., frustration regarding clients; vicarious trauma; Wilson, 2016) and/or organizationally desired emotions (e.g., staying neutral or calm; Bakker et al., 2008, 2010). Relationship with colleagues concerns the team atmosphere, including whether a respondent can rely on co-workers for help and social support. Lastly, information refers to the availability of feedback on respondents’ work performance. Lequeurre et al. (2013) used four items to measure each dimension. The Cronbach’s alpha was high, above 0.80 for each dimension (workload, 0.84; emotional load, 0.83; relationship with colleagues, 0.87; information, 0.86). All items were rated on a seven-point Likert scale ranging from 1 (never) to 7 (always). Higher scores in each item indicated higher levels of job demands or job resources. The total of item scores ranged from 4 to 28 for each dimension. The Cronbach’s alpha was 0.87 for all 16 items in this study. For each individual dimension, the Cronbach’s alpha values were 0.80, 0.68, 0.87, and 0.89 (workload, emotional load, relationship with colleagues, and information, respectively). We calculated the score of JD by summing up the item responses under workload and emotional workload. Meanwhile, we calculated JR by totaling item responses to the items in the relationships with colleagues and information subscales.

Several demographic and socioeconomic characteristics of the respondents were measured and included in our analytic model. These variables included gender (female = 1, male = 0), age (continuous, 20–50), marital status (never married = 1, other = 0), education (college degree or above = 1, below college education = 0), and social work license (yes = 1, no = 0).

### Analytical Approach

We conducted descriptive and correlation analyses to first observe the sample characteristics and correlations among all variables. Then, we conducted ordinary least squares (OLS) regression analysis to estimate associations between JD, JR, burnout, and psychological distress and to test whether the above associations are mediated by burnout and moderated by gender and age (Hayes, 2017). Structural equation modeling (SEM) can also be used to test the mediation and moderation effects. We conducted SEM analysis, and the results (available upon request) were not different from the regression approach. The framework underlying this study posits that the extent of burnout and the extent of psychological distress experienced by social workers are each determined by JD-R and social workers’ demographic characteristics. The specification of the analytic model is represented by the following equation:


$$Y_{i}=\alpha_{i}+\beta_{1}*\chi_{i}+\varepsilon_{i}$$

where *Y*i is burnout or psychological distress of the subject i; αi is the individual constant; χ is a vector of JD-R and the demographic characteristics of subject i; β is a vector of regression coefficients; and εi is the cross-sectional error component. For the model, in which psychological distress was the dependent variable, psychological distress was regressed onto burnout. All analyses were conducted using STATA software 16.0.

## Results

Table 1 presents the descriptive statistics of the variables. On average, the sample had a psychological distress score of 7.2, with a SD of 5.2. About 12% of social workers scored within the range for high psychological distress (i.e., above 13), and another 29% scored within the range for moderate psychological distress (i.e., between 8 and 12). In other words, 40% of the social workers sampled responded that they had experienced either moderate or high psychological distress within the past 30 days of completing the survey. Burnout scores averaged 53.9, with a SD of 16.5. The sample reported relatively high JD (*M* = 38.5, *SD* = 6.5) and JR (*M* = 40.8, *SD* = 7.0), on scales that ranged 8–56. This suggested that while the social workers experienced high JD at work, they also had access to many JR through support from the colleagues and available information at their agency.

**Table 1:** Descriptive statistics of key variables.

	Mean (S.D.)
1. Psychological stress (0–24)	7.2 (5.2)
High (13–24) [%]	11.6
Moderate (8–12) [%]	28.9
Low (0–7) [%]	59.5
2. Burnout (17–110)	53.9 (16.5)
3. Job demands (8–56)	38.5 (6.5)
4. Job resources (8–56)	40.8 (7.0)
5. Female [%]	78.2
6. Age (20–50)	31.8 (7.3)
7. Never married [%]	37.8
8. Education – College degree or above [%]	54.6
9. Social work license [%]	52.3

*N* = 897. Numbers in brackets show ranges of the variables.

The results of correlation analyses, shown in Table 2, were largely consistent with our hypotheses. JD was positively correlated with burnout (*r* = 0.18, *p* < 0.001), while JR was negatively correlated with burnout (*r* = −0.37, *p* < 0.001). JD and burnout were positively correlated with psychological distress (*r* = 0.15 and 0.52, respectively; both *p* < 0.001), while JR was negatively correlated with psychological distress (*r* = −0.25, *p* < 0.001). Age was negatively correlated with psychological distress and burnout. We did not find a significant correlation between gender and psychological distress. However, women in the sample tended to have low JD and burnout.

**Table 2:** Correlation analysis of key variables.

	1	2	3	4	5	6
1. Psychological stress	---					
2. Burnout	0.52^***^	---				
3. Job demands	0.15^***^	0.18^***^	---			
4. Job resources	−0.25^***^	−0.37^***^	0.30^***^	---		
5. Female	−0.04	−0.09^*^	−0.11^**^	−0.05	---	
6. Age	−0.14^***^	−0.17^***^	0.02	0.06	−0.04	---

*N* = 897.

* *p* < 0.05;

** *p* < 0.01;

*** *p* < 0.001.

Table 3 presents the standardized estimates of burnout. The adjusted R-square of the model was 0.25. JD and JR had significant associations with burnout. A one SD increase in JR was associated with a decrease of 0.45 SDs in psychological distress. Meanwhile, an increase of one SD in JD was associated with an increase of 0.30 SDs in psychological distress. These findings support hypothesis 1. Female and older social workers reported low burnout (*B* = −0.07, *p* < 0.05 and *B* = −0.10, *p* < 0.01, respectively).

**Table 3:** Regression analysis of burnout.

	Beta	*b*	S. E.	*t*	*p*
Job demands	0.30	0.75	0.08	9.51	***
Job resources	−0.45	−1.06	0.07	−14.70	***
Female	−0.07	−2.98	1.19	−2.51	*
Age (20–50)	−0.10	−0.23	0.08	−2.77	**
Never married	0.05	1.79	1.24	1.44	
Education – College degree or above	0.04	1.21	1.05	1.15	
Social work license	−0.01	−0.06	1.01	−0.06	
Adjusted R-square	0.25				

*N* = 897. *F*(7, 889) = 42.89.

* *p* < 0.05;

** *p* < 0.01;

*** *p* < 0.001.

**Figure 1:**
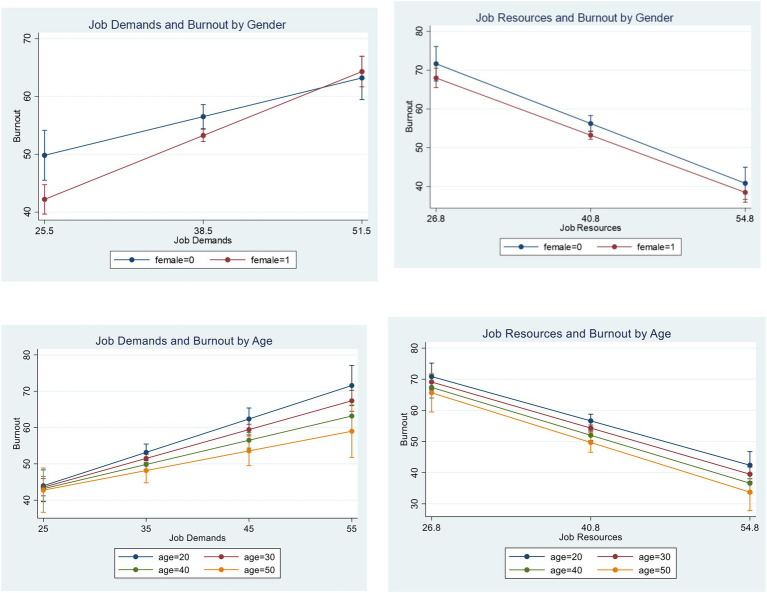
Effects of job demands and resources (JD-R) on burnout, by gender and age. The graph was based on results from Table 3.

To test the moderation effects of gender and age on the relations between JD-R and psychological distress, we added the interaction variable between each of JD and JR with gender or age, into the regression model. To avoid multilinearity, only one interaction variable was added into the regression each iteration. The results of the four interaction regressions (JD*gender, JD*age, JR*gender, and JR*age) are presented in Figure 1. The interaction effect of JD and female on psychological distress was statistically significant (*B* = 0.34, *p* < 0.05), suggesting that the effect of JD on psychological distress was stronger for female social workers than for male social workers. No other interaction effect was found among the other interaction terms.

Table 4 presents the standardized estimates of psychological distress. Two models are presented. The first model included JD-R, along with the demographic characteristics. Burnout was added into the second regression model. The adjusted R-square of Model 1 was 0.13, while the adjusted R-square of Model 2 was 0.29. Increasing JD by one SD was associated with a 0.23-SD increase in psychological distress, and increasing JR by one SD was associated with a 0.31-SD reduction in psychological distress. Age appeared to have a negative association with psychological distress (*B* = −0.13, *p* < 0.01).

**Table 4:** Regression analysis of psychological distress.

	Model 1			Model 2		
	Beta	S. E.	*p*	Beta	S. E.	*p*
Burnout	---	---		0.46	0.01	***
Job demands	0.23	0.03	***	0.10	0.03	**
Job resources	−0.31	0.02	***	−0.10	0.02	**
Female	−0.04	0.40		−0.01	0.37	
Age (20–50)	−0.13	0.03	**	−0.08	0.03	*
Never married	−0.02	0.43		−0.05	0.39	
Education – College degree or above	0.03	0.36		0.01	0.32	
Social work license	0.04	0.34		0.04	0.31	
Adjusted R-square	0.13			0.29		

*N* = 897.

* *p* < 0.05;

** *p* < 0.01;

*** *p* < 0.001.

In Model 2, burnout had a strong positive effect on psychological distress (*B* = 0.46, *p* < 0.001). After the inclusion of burnout in the model, the estimates of JD and JR reduced from 0.23 and −0.31 to about 0.10 and −0.10, respectively. These findings support hypothesis 2. After adding burnout into the model, the estimates of JD and JR both lowered substantially, suggesting that burnout is a mediator between JD, JR, and psychological distress. These findings confirm hypothesis 3.

**Figure 2:**
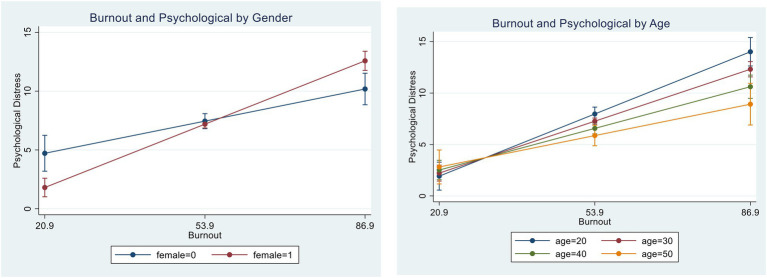
Effects of burnout on psychological distress, by gender and age. The graph was based on results from Table 4.

The moderation effects of gender and age on the relation between burnout and psychological distress were then tested, and the results of the two interaction regressions (burnout*gender, burnout*age) are presented in Figure 2. The interaction between burnout and gender achieved statistical significance (*B* = 0.08, *p* < 0.001), suggesting that burnout had a larger effect on psychological distress for female social workers than for male social workers. The interaction between burnout and age achieved statistical significance as well (*B* = −0.01, *p* < 0.05), suggesting that burnout had less of an effect on psychological distress among older social workers. These findings support hypothesis 4.

## Discussion

Findings of descriptive analysis indicated that about 41% of the sampled social workers experienced moderate to high psychological distress. As psychological distress is positively related to serious mental illness, alcohol and substance use, and negative work outcomes across cultural contexts (Furukawa et al., 2003; Hardy et al., 2003; Kessler et al., 2010), it is imperative to understand factors of psychological distress in social workers in China, as well as potential services or intervention to reduce this distress. The findings also show that social workers in China have high levels of burnout and JD. At the same time, they have relatively high JR available to them. Thus, the relations among JD, JR, burnout, and psychological distress are worthy of further examination.

Regression results first provided support for the hypothesized dual processes through which JD-R affect burnout in social workers in China. The health-impairment, or energy depletion, process was indicated by the positive association between JD and burnout, whereas the motivation process was indicated by JR’s negative association with burnout. Thus, social workers who can draw upon JR are less likely to experience burnout. The magnitude of the estimates show that JR have a greater effect on burnout in Chinese social workers than JD. Meanwhile, moderation analyses showed a moderation effect of gender, whereby JD had a greater effect on female social workers’ burnout than on male’s counterparts.

The regression results for psychological distress showed that JD-R and burnout are important predictors of the social workers’ psychological distress. Both estimates of JD-R, however, decreased substantially once burnout was included in Model 2, suggesting that burnout may mediate the relation between JD-R and psychological distress. Moderation analyses showed that the effects of burnout on psychological distress were moderated by gender and age. Female social workers with high burnout were more likely to have greater psychological distress than their male counterparts. Likewise, younger social workers with high burnout were more likely to have greater psychological distress than their older counterparts.

Taken together, the results are in line with and expand upon previous findings from other studies’ application of the JD-R model to samples of professionals from other occupational groups (Demerouti et al., 2001; Bakker et al., 2003; Hakanen et al., 2008). This study expands past literature by providing support for the application of JD-R in studying burnout and subsequent psychological distress in social workers in China.

The findings of this study have practice, policy, and research implications. With respect to practice, given that this sample reported, on average, high JD, and given the positive relations between JD and burnout and JD and psychological distress, employers of social workers in China need to be cognizant of the JD projected onto their workers. More importantly, however, employers must make supportive services available to their social work employees to mitigate the effects of JD on burnout and psychological distress. In the current study, the sample of social workers reported high JR. Yet, it is imperative that these numbers are understood with respect to the urban context of these social workers. As previously mentioned, social work in China has developed rather unevenly, as resource availability varies by locale. Given that Chengdu is the capital of Sichuan province and one of the three most populous cities in western China, the relatively high availability of JR to the social workers in the study sample is consistent with past literature regarding the rapid development of social work in urban areas (Liu et al., 2020). Employers of social workers, ranging from social service agencies to hospitals and community centers, need to maintain the availability of sufficient JR to their workers. This may be a challenge for agencies that have limited resources to sustain their organization and practice, necessitating that federal, provincial, and local policies direct funding and resources to smaller agencies to support employees with burnout-reducing JR. For example, the strong positive effect of burnout on psychological distress suggests that reducing burnout can also reduce psychological distress and potential severe mental illness (Furukawa et al., 2003; Kessler et al., 2010). There is therefore a need to implement supportive services and interventions in organizations that employ social workers to buffer the effects of JD on burnout and psychological distress. For example, empirical studies have shown that mindfulness-based stress reduction (MBSR), mindfulness-based cognitive therapy (MBCT), and mindfulness-based interventions (MBI) all can effectively reduce psychological distress and promote mental health and well-being (Zoogman et al., 2014; Lomas et al., 2018; Guidetti et al., 2019; Suleiman-Martos et al., 2020). In fact, MBSR and MBCT have specifically been implemented as interventions for employees such as physicians, teachers, and psychotherapists, and related outcomes included reduced emotional exhaustion and psychological distress (see Janssen et al., 2018 for review). In one study that tested the effects of MBSR on nurses, another helping profession, treatment group participants had significantly lower Maslach Burnout Inventory scores than their control counterparts (Cohen-Katz et al., 2005), highlighting the potential of implementing such interventions in the Chinese social worker population. The findings of moderation analyses in this study call for gender- and age-sensitive adaptations to such interventions and services for social workers in China.

The findings of this study also have implications for future research. These results provide support for further examination of the relations among JD-R, burnout, and psychological distress, as all three concepts contain multiple dimensions and may be measured and operationalized differently. JD-R can have multiple dimensions of JD and JR. For example, Demerouti et al. (2001) identified five JD dimensions (physical workload, time pressure, recipient contact, physical environment, and shift work) and six JR dimensions (feedback, rewards, job control, participation, job security, and supervisor support). Lequeurre et al. (2013), on the other hand, identified seven JD dimensions (pace and amount of work, mental load, emotional load, physical efforts, changes in tasks, ambiguities about work, and uncertainty about the future) and seven JR dimensions (information, communication, participation, relationship with colleagues, relationship with superior, remuneration, and independence in the work). Due to resource constraints, we only focused on two dimensions of each JD and JR, specifically those that have previously shown significant effects in other studies (Lequeurre et al., 2013). Future studies may examine the extent to which other JD-R dimensions affect burnout and psychological distress. Likewise, burnout and psychological distress can include multiple dimensions. It is likely that JD-R components may differentially affect the various dimensions of burnout and psychological distress each, warranting future studies that measure these multidimensional constructs more comprehensively.

The findings and implications of the present study must be considered within several limitations. First, our analyses were based on a cross-sectional dataset, which can only approximate associative relations among JD-R, burnout, and psychological distress. Future research can use a longitudinal design to better approximate the causal relations of these variables. Second, unobserved variables, which were not included in this study, could have had effects on JD-R, burnout, and psychological distress. Such variables may include job type and personal traits. While all respondents were social workers, the roles that social workers play across China vary greatly. Our sample may have consisted of direct practitioners, who work closely with vulnerable populations, as well as administrative staff, who work within the bounds of macro-level practice (e.g., policy and advocacy). Further, psychological health is affected by personal traits like mindfulness, grit, and resilience (Zoogman et al., 2014; Suleiman-Martos et al., 2020). These were not measured in our study and may explain variations in social workers’ psychological distress. The absence of these unobserved variables may have effects on the estimates reported in this study. Third, data gathered on JD-R, burnout, and psychological distress were from self-reports of the subjects. Self-reporting leaves our data subject to unintended and intended reporting errors. Social desirability bias, for example, may lead respondents to underreport their JD and psychological distress, while overreporting JR. Future studies might consider using data triangulation through colleague reports and employer reports. Finally, the findings of this study are based on data from social workers in two districts in Chengdu, the capital city of Sichuan province in China. While the sample size and response rate support our confidence in these results, the extent to which these findings can be generalizable to all Chinese social workers is unknown and requires further research.

## Conclusion

This study analyzed data collected from 897 social workers in Chengdu, China, to investigate the extent to which JD-R affects burnout and psychological distress in social workers in China. It also investigated whether these relations are moderated by gender and age. The findings of this study support past findings from cross-cultural research, which have indicated that JD-R affects burnout and health outcomes through a dual process. The findings expand upon this research by providing evidence to support JD-R’s dual process in a sample of Chinese social workers. The results underscore the importance of reducing JD and increasing JR for social workers in China, an emerging profession with a high turnover rate. They also emphasize that burnout may serve as a mediator in the relation between JD-R and psychological distress, necessitating gender- and age-sensitive interventions to mitigate burnout and psychological distress among social workers in China.

## Data Availability Statement

The raw data supporting the conclusions of this article will be made available by the authors, without undue reservation.

## Ethics Statement

The studies involving human participants were reviewed and approved by Review Committee, Research Institute of Social Development, Southwestern University of Finance & Economics. Written informed consent for participation was not required for this study in accordance with the national legislation and the institutional requirements.

## Author Contributions

XX and CH: conceptualization and resources. XX, CH, SC, YZ, and JF: methodology and software, validation, formal analysis, and writing – original draft preparation. XX, CH, YZ, and JF: investigation and data curation. All authors contributed to the article and approved the submitted version.

## Funding

This paper was supported by the National Social Science Fund (No. 16BZZ058).

## Conflict of Interest

The authors declare that the research was conducted in the absence of any commercial or financial relationships that could be construed as a potential conflict of interest.

## Publisher’s Note

All claims expressed in this article are solely those of the authors and do not necessarily represent those of their affiliated organizations, or those of the publisher, the editors and the reviewers. Any product that may be evaluated in this article, or claim that may be made by its manufacturer, is not guaranteed or endorsed by the publisher.
